# Metabolomic-derived endotypes of age-related macular degeneration (AMD): a step towards identification of disease subgroups

**DOI:** 10.1038/s41598-024-59045-z

**Published:** 2024-05-27

**Authors:** Kevin Mendez, Ines Lains, Rachel S. Kelly, João Gil, Rufino Silva, John Miller, Demetrios G. Vavvas, Ivana Kim, Joan Miller, Liming Liang, Jessica A. Lasky-Su, Deeba Husain

**Affiliations:** 1 Retina Service, Massachusetts Eye and Ear, Harvard Medical School, 243 Charles Street, Boston, MA 02114 USA; 2 Channing Division of Network Medicine, Department of Medicine, Brigham and Women’s Hospital and Harvard Medical School, Boston, MA USA; 3 Centre for Integrative Metabolomics & Computational Biology, School of Science, Edith Cowan University, Perth, WA Australia; 4 Faculty of Medicine, University of Coimbra, Coimbra, Portugal; 5 Department of Ophthalmology, Coimbra Hospital and University Center, Coimbra, Portugal; 6 Association for Innovation and Biomedical Research in Light and Image (AIBILI), Coimbra, Portugal; 7 Program in Genetic Epidemiology and Statistical Genetics, Department of Epidemiology, Harvard T.H. Chan School of Public Health, Boston, MA USA; 8 Department of Biostatistics, Harvard T.H. Chan School of Public Health, Boston, MA USA

**Keywords:** Age-related macular degeneration, AMD, Metabolomics, Endotyping, Metabo-endotypes, Translational research, Diagnostic markers

## Abstract

Age-related macular degeneration (AMD) is a leading cause of blindness worldwide, with a complex pathophysiology and phenotypic diversity. Here, we apply Similarity Network Fusion (SNF) to cluster AMD patients into putative metabolomics-derived endotypes. Using a discovery cohort of 163 AMD patients from Boston, US, and a validation cohort of 214 patients from Coimbra, Portugal, we identified four distinct metabolomics-derived endotypes with varying retinal structural and functional characteristics, confirmed across both cohorts. Patients clustered into Endotype 1 exhibited a milder form of AMD and were characterized by low levels of amino acids in specific metabolic pathways. Meanwhile, patients clustered into both Endotype 3 and 4 were associated with more severe AMD and exhibited low levels of fatty acid metabolites and elevated levels of sphingomyelins and fatty acid metabolites, respectively. These preliminary findings indicate that metabolomics-derived endotyping may offer a refined strategy for categorizing AMD patients based on their specific pathophysiological underpinnings, rather than relying solely on traditional observational clinical indicators.

## Introduction

Age-related macular degeneration (AMD) is a leading cause of adult blindness worldwide, predicted to affect 288 million individuals by 2040^1^. In a clinical setting, AMD is commonly classified into two main subtypes: exudative or "wet" AMD, which is characterized by the development of new vessels that bleed and leak fluid into the retina; and non-exudative or "dry" AMD, in which these features are absent^2,3^. Non-exudative AMD is generally further subdivided into early and intermediate forms, which are marked by the presence of drusen and pigmentary changes, and a late form characterized by the loss of chorioretinal tissue (i.e. geographic atrophy)^1^.

Recent advances in retinal imaging have highlighted the extensive phenotypic variability of AMD, and multiple clinicians and researchers have suggested that different phenotypes may have distinct underlying mechanisms and prognoses^4^. It is currently hypothesized that the early and intermediate forms of AMD might not represent a single disease, but rather a collection of subtypes that have different risks of progression to the advanced forms. Because of this, there are currently no treatments available for the early and intermediate dry forms of the disease which constitute majority of the cases of AMD. This suggests the possible existence of multiple AMD endotypes (i.e. subtypes of disease defined by functional or pathophysiologic mechanisms) that have clinically significant differences in patient outcomes. Unraveling these endotypes and their underlying pathogenesis is critical and may contribute to development of new therapies for dry AMD and, importantly, to halt its progression to the vision-impairing forms of the disease.

The current limited understanding of the multiplicity of AMD presentations likely stems from its multifactorial nature, involving a complex interplay between genetic and environmental risk factors^5^. The relative contribution of these factors to the formation of AMD endotypes likely varies but remains unknown. Metabolomics, the qualitative and quantitative analysis of metabolites (< 1–1.5 KDa), captures the cumulative effects of the genome and its interaction with environmental exposures. As a result, the metabolome is believed to closely relate to disease phenotype^6^, and offers a novel and promising approach for identifying endotypes, as demonstrated in other multifactorial diseases, such as asthma^7,8^.

Multiple studies have reported that the plasma metabolomic profile of patients with AMD differs with disease severity and is associated with AMD progression^9,10^. However, no studies have yet employed an untargeted approach utilizing the global metabolome to identify endotypes in AMD. This study aims to derive putative clinically meaningful metabolomic-derived endotypes (metabo-endotypes) of AMD and validate them in a separate cohort, with the ultimate goal of contributing to a better understanding of the underlying mechanisms of different AMD presentations, which could lead to the identification of potential treatment targets.

## Methods

### Study design

This is a cross-sectional study that is part of a prospective, observational project in AMD metabolomics that is taking place at the Massachusetts Eye and Ear (MEE), Harvard Medical School in Boston, US, and the Faculty of Medicine of the University of Coimbra (FMUC) in Coimbra, Portugal, in collaboration with the Association for Innovation and Biomedical Research on Light and Image (AIBILI) and the "Centro Hospitalar e Universitário de Coimbra". The clinical protocol adhered to the Health Insurance Portability and Accountability Act (HIPAA) guidelines and the principles of the Declaration of Helsinki. Approval for the study was obtained from the Institutional Review Boards of MEE, FMUC, AIBILI, and the Portuguese National Data Protection Committee (CNPD).

### Study population

Our study population was derived from two distinct locations, as previously reported by our group^11–14^. Participants were enrolled at MEE, Boston, US, and in FMUC/AIBILI in Coimbra, Portugal. All participants provided written informed consent before taking part in the study.

At both study sites, we recruited individuals diagnosed with AMD and control subjects aged 50 years or older who exhibited no signs of AMD. Several exclusion criteria were applied. These included the presence of any other vitreoretinal disease, active uveitis or ocular infection, significant media opacities that hindered ocular fundus examination, refractive errors of 6 diopters or more of spherical equivalent, a history of retinal surgery, and any ocular surgery or intraocular procedure (such as laser treatment and intraocular injections) conducted within 90 days prior to enrollment. Furthermore, individuals diagnosed with diabetes mellitus were also excluded from the study.

### Study protocol

All participants provided a comprehensive medical history and underwent a complete bilateral ophthalmologic examination. In addition, non-stereoscopic color fundus photographs (CFP) were taken using either a Topcon TRC-50DX (Topcon Corporation, Tokyo, Japan) or a Zeiss FF-450Plus (Carl Zeiss Meditec, Dublin, CA) camera. Spectral-domain OCT (SD-OCT, Spectralis^®^, Heidelberg, Germany) imaging was also performed on the subjects.

To obtain plasma samples for metabolomic analysis, venous blood was collected between 7:30 and 9:00 AM from the participants in sodium-heparin tubes after confirming overnight 8-h fasting. These samples were centrifuged within 30 min (1500 rpm, 10 min, 20 °C) to separate the plasma. Plasma aliquots of 1.5 mL were then transferred to sterile cryovials and stored at − 80 °C for future analysis. For patients who were recruited during regular ophthalmic appointments, an additional visit was scheduled within a maximum of one month after study inclusion to collect blood samples, ensuring overnight fasting was maintained.

All data collected during the study were securely stored using REDCap electronic data capture tools to maintain the integrity and confidentiality of the participants' information.

### AMD diagnosis and staging

To ensure consistency in AMD diagnosis and staging, images were standardized using software developed by our group^15^ prior to grading. Two out of three independent experienced graders analyzed field 2 color fundus photography (CFP) according to the age-related eye disease study (AREDS) classification system^16,17^. In cases of disagreement, a senior author (RS or DH) determined the final categorization. Following the most recent AREDS2 definitions^17^ and building upon previous reports^12,13,18^, we established the following groups based on the AREDS classification system:

1. Early AMD – identified by drusen with a maximum size greater than or equal to C0 but smaller than C1, or the presence of AMD characteristic pigment abnormalities in the inner or central subfields.
2. Intermediate AMD – marked by the presence of drusen with a maximum size greater than or equal to C1, or drusen with a maximum size greater than or equal to C0 if the total area occupied is greater than I2 for soft indistinct drusen and greater than O2 for soft distinct drusen.
3. Late AMD – characterized by the presence of GA according to the criteria described above (GA or "dry" late AMD) or evidence of neovascular AMD (choroidal neovascularization, CNV or "wet" AMD).

### Ocular coherence tomography grading

For Ocular Coherence Tomography (OCT) grading, two out of four independent investigators, who were masked to clinical data, analyzed all OCT images to determine the presence or absence (dichotomous variable) of various features. These features included: ellipsoid zone (EZ) disruption; classic drusen [defined as subretinal pigment epithelium (RPE) deposits; presence of ≥ 1 drusen was graded as "yes"]; subretinal drusenoid deposits (SDD)14 (presence of ≥ 1 was graded as "yes"); hyperreflective foci (presence of ≥ 1 was graded as "yes"); retinal atrophy (defined as increased light transmission to the choroid and loss of external retinal layers15); fibrosis; choroidal neovascularization; subretinal and intraretinal fluid; and serous pigment epithelium detachment (PED).

In cases of disagreement, a senior author intervened to resolve the discrepancies and determine the final grading for the OCT images.

### Sample collection and mass spectrometry analysis

After all subjects had been recruited, plasma samples from Coimbra, Portugal were shipped to MEE in dry ice (through TNT^®^ Express, US, INC). Subsequently, all samples from both study locations were sent to Metabolon, Inc^®^, also in dry ice (through TNT®Express, US, INC). In both cases, samples arrived frozen in less than 48 h and were immediately stored at − 80 °C until processing.

Metabolon, Inc^®^ performed non-targeted mass spectrometry (MS) analysis using ultra-high-performance liquid chromatography-tandem MS (UPLC-MS/MS). The process involved four ionization methods: (1) ‘PosEarly’ for hydrophilic compounds, (2) ‘PosLate’ for hydrophobic compounds, both using a Waters UPLC BEH C18 column; (3) ‘Neg’ with basic negative ion conditions on a dedicated C18 column; and (4) ‘Polar’ with negative ionization on a Waters UPLC BEH Amide column, with each method followed by MS and MSn scans^19^. To account for potential batch effects and variations resulting from instrument inter-day differences, Metabolon, Inc^®^ performed data normalization according to their standard protocols^14^. In brief, each compound was corrected in run-day blocks by registering the medians to equal one (1.00) and normalizing each data point proportionately (i.e. in our study, 2 days).

To merge the pilot and newer datasets, we performed normalization by dividing each dataset by the median of the control samples for that study, then median scaled^14^. This approach ensured the accuracy and consistency of the MS analysis across all samples.

### Dark adaptation

Dark adaptation (DA), a test to assess retinal function, was performed on patients from the Boston, US population. To avoid prior light exposure, DA was performed on a separate day than retinal imaging, within a maximum time limit of one month after enrolling in the study. Our protocol has been described previously in detail^6^. Briefly, we evaluated DA using the AdaptDx^®^ dark adaptometer (MacuLogix, Harrisburg, PA) extended protocol (20 min)^5^. Sensitivity was estimated using a modified staircase threshold estimate procedure, with an initial stimulus intensity of 5 scot cd/m^2^. The test ended when the patient’s sensitivity recovered by 3.0 log units (corresponding to the level of 5 × 10^–3^ scot cd/m^2^) or the test duration reached 20 min, whichever came first. The machine then estimates the slope of the second component of rod-mediated dark adaptation and extrapolates the time required for the sensitivity to recover by 3.0 log units, which is designated as rod-intercept time (RIT). For analysis, RIT data was exported and eyes with fixation errors ≥ 30% were excluded. Additionally, data on successive threshold measurements was exported to calculate the area under the dark adaptation curve (AUDAC).

### Statistical methods

#### Derivation of “metabo-endotypes” in Boston, US

We grouped the 163 subjects from the Boston cohort into distinct putative metabolomic-driven endotypes (metabo-endotypes) based on their metabolite residuals. This was performed utilizing the Similarity Network Fusion (SNF) R package: SNFtool version 2.2 and spectral clustering. SNF is a patient-centered approach that integrates 'omic' data through the construction and merging of patient networks^20,21^. We treated each of the four metabolite platforms as separate 'omics', building a network for each platform. The distribution of these metabolites across the ionization modes is as follows: Pos Early (151 metabolites), Pos Late (199 metabolites), Polar (65 metabolites), and Negative (364 metabolites). These networks were then fused using the network fusion step of SNF, a non-linear method based on message-passing theory^22^. SNF parameters were set according to the recommended setting by Wang, et al.^20^ with k = 54 and alpha = 0.8. The k value was computed using the recommended algorithm n/c, where n is the number of participants and c is the expected number of clusters^20^. We hypothesized c = 3 AMD endotypes based on standard AMD classifications: Early, Intermediate, and Late AMD. We then employed spectral clustering^23^ on both platform-specific networks and the fused network to identify metabolomic-driven clusters within each platform. To determine the optimal number of clusters for each platform, we utilized the rotational cost approach, as recommended in the SNF package, by Wang et al.^20^. In the rotational cost method, the optimal cluster quantity is derived soley by minimizing the cost-function value across all possible rotations to achieve the most effective alignment of eigenvectors with the standard coordinate system^24^. The evaluation range for identifying the optimal cluster count via the rotational cost method was set to span from 2 to 20.

#### Exploration clinical characteristics of metabo-endotypes

To explore whether there were measurable clinical or functional differences between the groups of individuals in each metabolomic-derived endotype, we employed one-way analysis of variance (ANOVA) for continuous variables and chi-squared tests for categorical variables.

#### Validation of metabo-endotypes in Coimbra, Portugal

We employed the label propagation classifier approach, a graph-based semi-supervised machine learning method, to predict metabo-endotypes (as defined in Boston) for the Coimbra, Portugal population^25^. To utilize this approach, we first constructed a similarity matrix using SNF, combining the Boston, US and Coimbra, Portugal populations based on their metabolite residual data. Subsequently, we applied the classifier to assign each of the Portugal validation subjects to one of the Boston, US-defined metabo-endotypes.

Next, we assessed clinical and phenotypic characteristics between the Portuguese subjects metabo-endotypes, following the same approach as for Boston. We considered the metabo-endotypes as validated if the clinical characteristics that differentiated the AMD metabo-endotypes generated in Boston, US also differentiated the AMD metabo-endotypes in Coimbra, Portugal.

#### Identification of metabolomic drivers of meta-endotypes

To identify the metabolites with the greatest contribution to the formation of each metabolomic-endotype, we employed analysis of variance (ANOVA) and subsequent Tukey Honest Significant Differences post-hoc pairwise comparisons. To generate a single effect-estimate for the contribution of each metabolite to the formation of the metabo-endotypes, we meta-analyzed the results from both the Boston, US and Coimbra, Portugal study populations using the R package 'metap' [version 1.8]. Our analysis involved a two-step approach: first, we restricted the metabolites based on an ANOVA q-value < 0.05; then, we further refined the selection by applying a q-value < 0.05 for each additional post-hoc comparison against each endotype. Q-values were derived from the Benjamini–Hochberg procedure.

## Results

### Study population

We included a total of 377 subjects: 163 from Boston, US and 214 from Coimbra, Portugal. The clinical and demographic characteristics of these subjects are presented in Table 1. The mean age of participants from Boston was lower than those from Coimbra, Portugal (72.7 vs. 77.2 years, P < 1e–04), with comparable BMI and sex distributions. There were fewer early (15.0% vs. 33.7%) and more intermediate AMD stages in Portugal (59.8% vs. 38.7%, P < 1e-04), along with a higher proportion of non-smokers (84.1% vs. 51.5%, P < 1e–04).

**Table 1:** Demographics table of Boston and Portugal.

	Boston (n = 163)	Portugal (n = 214)	P-value
Age, mean ± SD	72.7 ± 7.6	77.2 ± 7.9	< 1e-04
Female sex, n (%)	113 (69.3)	135 (63.1)	0.25
BMI, mean ± SD	27.1 ± 4.8	27.2 ± 4.4	0.78
Race, n (%)			0.18
White	156 (95.7)	211 (98.6)	
Black	0 (0.0)	3 (1.4)	
Hispanic	5 (3.1)	0 (0.0)	
Asian	2 (1.2)	0 (0.0)	
Smoking, n (%)			< 1e–04
Non-smoker	84 (51.5)	180 (84.1)	
Ex-smoker	76 (46.7)	33 (15.4)	
Smoker	3 (1.8)	1 (0.0)	
AMD stage, n (%)			< 1e–04
Early	55 (33.7)	32 (15.0)	
Intermediate	63 (38.7)	128 (59.8)	
Late	45 (27.6)	54 (25.2)	

*BMI* body mass index, *SD* standard deviation. P value is based on the ANOVA test for continuous variables and the chi-square test for categorical variables.

### Boston, US metabo-endotypes

We employed SNF to integrate the networks from the four platforms, achieving convergence after 10 iterations, and subsequently performed spectral clustering. This analysis revealed that the optimal division was into four distinct clusters, comprising 33, 43, 47, and 40 AMD cases, respectively. These clusters were subsequently identified as the AMD metabo-endotypes.

**Figure 1:**
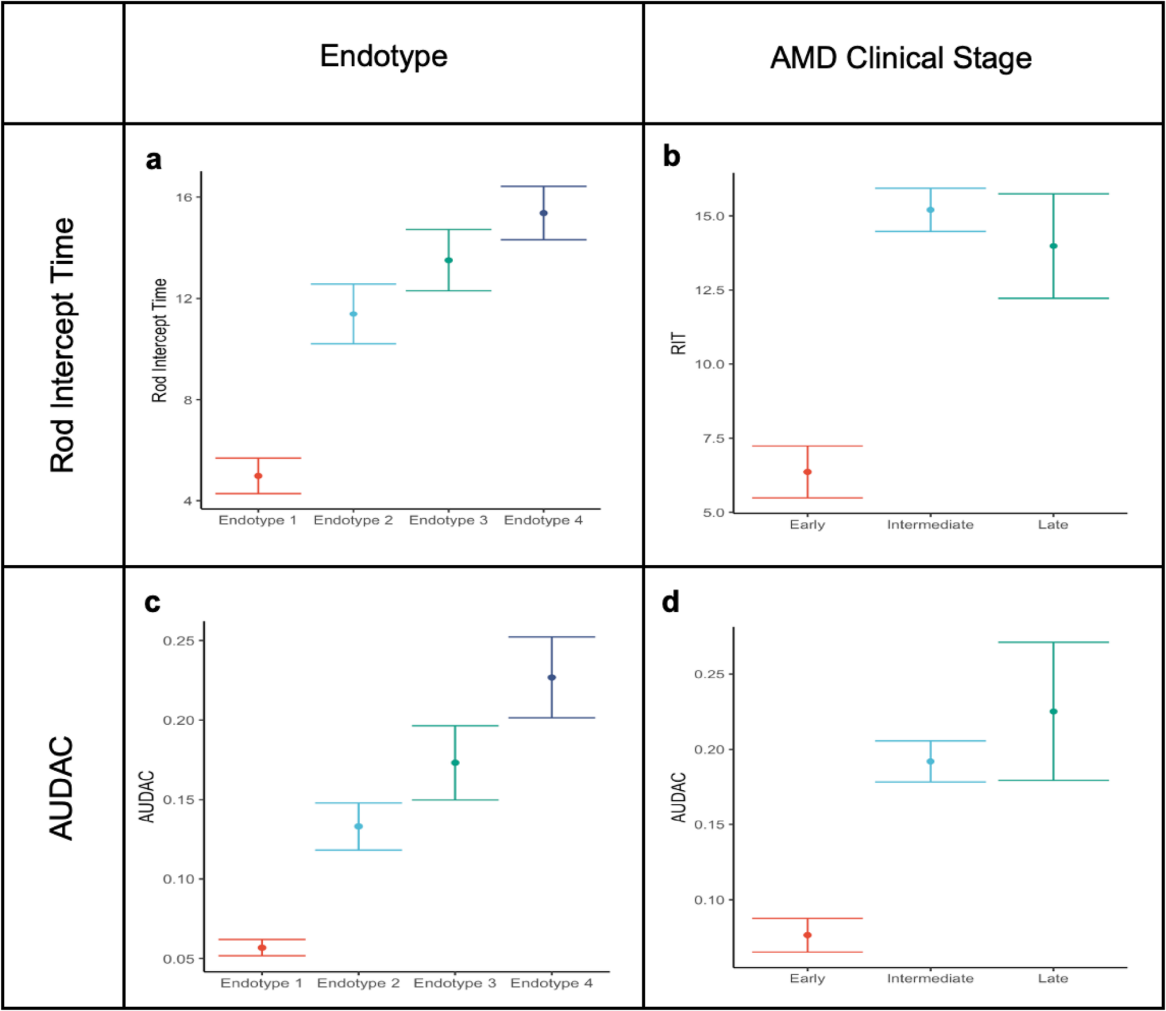
Mean and standard error of rod intercept time by (**a**) AMD Metabo-endotypes and (**b**) AMD clinical stage, and AUDAC by (**c**) AMD metabolomic-endotype and (**d**) AMD clinical stage.

The associations between the four endotypes and various clinical and demographic factors were assessed (Table 2). No significant associations were found with demographic factors such as age, sex, BMI, race, and smoking. Individuals within the different metabo-endotypes differed with respect to rod intercept time (RIT) (p = 0.001) and AUDAC (p = 0.001). As shown in Fig. 1, a clear difference in retinal function was observed across metabo-endotypes (values in mean ± SD), with Endotype 1 showing the most normal results (RIT, 5.0 ± 1.7; AUDAC, 0.06 ± 0.01), and endotypes 2 (RIT, 11.4 ± 6.6; AUDAC, 0.13 ± 0.06), 3 (RIT, 13.5 ± 6.4; AUDAC, 0.17 ± 0.12), and 4 (RIT, 15.4 ± 5.7; AUDAC, 0.23 ± 0.14) becoming progressively more affected (Fig. 1, Table 2).

**Table 2:** Clinical and demographic association with the 4 endotypes.

	Endotypes (B = Boston P = Portugal)									
	1 (B)	2 (B)	3 (B)	4 (B)	1 (P)	2 (P)	3 (P)	4 (P)	P-value (B)	P-value (P)
Demographics										
Age, mean ± SD	72.2 ± 7.6	71.7 ± 8.3	72.7 ± 7.9	74.3 ± 6.5	77.9 ± 7.2	77.1 ± 7.5	77.5 ± 8.0	76.1 ± 8.1	0.448	0.791
Female sex, n (%)	23 (69.7)	31 (72.1)	31 (66.0)	28 (70.0)	8 (72.7)	29 (59.2)	72 (62.1)	26 (68.4)	0.937	0.735
BMI, mean ± SD	27.3 ± 6.0	27.8 ± 5.4	26.4 ± 3.4	27.0 ± 4.5	26.7 ± 3.9	27.4 ± 5.3	26.9 ± 4.3	28.2 ± 3.6	0.621	0.449
Race, n (%)									0.521	0.158
White	28 (96.6)	18 (94.7)	24 (100.0)	10 (100.0)	11 (100.0)	49 (0.0)	115 (99.1)	36 (94.7)		
Black	0 (0.0)	0 (0.0)	0 (0.0)	0 (0.0)	0 (0.0)	0 (0.0)	1 (0.9)	2 (5.3)		
Hispani	1 (3.4)	0 (0.0)	0 (0.0)	0 (0.0)	0 (0.0)	0 (0.0)	0 (0.0)	0 (0.0)		
Asian	0 (0.0)	1 (5.3)	0 (0.0)	0 (0.0)	0 (0.0)	0 (0.0)	0 (0.0)	0 (0.0)		
Smoking, n (%)									0.07	0.777
Non-smoker	24 (72.7)	20 (46.5)	25 (53.2)	15 (37.5)	11 (100.0)	42 (85.7)	95 (81.9)	32 (84.2)		
Ex-smoker	9 (27.3)	20 (46.5)	22 (46.8)	25 (62.5)	0 (0.0)	7 (14.3)	20 (17.2)	6 (15.8)		
Smoker	0 (0.0)	3 (7.0)	0 (0.0)	0 (0.0)	0 (0.0)	0 (0.0)	1 (0.9)	0 (0.0)		
AMD stage, n (%)									4.53e–07	0.02
Early	22 (66.7)	5 (11.6)	10 (21.3)	18 (45.0)	2 (18.2)	9 (18.4)	17 (14.7)	4 (10.5)		
Intermediate	6 (18.2)	28 (65.1)	16 (34.0)	13 (32.5)	8 (72.7)	37 (75.5)	61 (52.6)	22 (57.9)		
Late	5 (15.2)	10 (23.3)	21 (44.7)	9 (22.5)	1 (9.1)	3 (6.1)	38 (32.8)	12 (31.6)		
Retinal function										
RIT, mean ± SD	**5.0 ± 1.7**	**11.4 ± 6.6**	**13.5 ± 6.4**	**15.4 ± 5.7**	–	–	–	–	**0.001**	–
AUDAC, mean ± SD	**0.06 ± 0.01**	**0.13 ± 0.06**	**0.17 ± 0.12**	**0.23 ± 0.14**	–	–	–	–	**0.001**	–
Retinal structure										
Classic drusen, n (%)	23 (39.0%)	62 (79.5%)	66 (72.5%)	62 (83.8%)	14 (66.7%)	59 (60.8%)	147 (67.1%)	49 (69.0%)	3.29e–08	0.67
Reticular drusen subretinal drusenoid deposits, n (%)	26 (42.6%)	35 (44.9%)	41 (45.1%)	36 (48.6%)	10 (47.6%)	57 (58.8%)	138 (63.3%)	50 (70.4%)	0.91	0.21
Atrophy, n (%)	**4 (6.2%)**	**20 (25.6%)**	**30 (33.0%)**	**22 (29.7%)**	**1 (4.8%)**	**7 (7.1%)**	**42 (18.8%)**	**12 (16.9%)**	**0.0009**	**0.03**
EZ disruption, n (%)	**9 (14.3%)**	**37 (47.4%)**	**55 (60.4%)**	**51 (68.9%)**	**5 (23.8%)**	**34 (35.1%)**	**107 (47.8%)**	**33 (46.5%)**	**3.65e-10**	**0.045**
Hyperreflective foci, n (%)	**4 (6.2%)**	**19 (24.4%)**	**30 (33.0%)**	**38 (51.4%)**	**4 (19%)**	**28 (28.9%)**	**95 (44.0%)**	**29 (41.4%)**	**1.19e–07**	**0.02**
Drusenoid PED, n (%)	2 (11.1%)	5 (6.7%)	10 (12.2%)	10 (13.5%)	–	–	–	–	0.56	–
Serous PED, n (%)	1 (1.8%)	3 (5.8%)	2 (3.0%)	1 (2.1%)	0 (0.0%)	6 (6.2%)	12 (5.7%)	4 (5.6%)	0.65	0.72
Subretinal fluid, n (%)	2 (3.2%)	9 (11.5%)	2 (2.2%)	4 (5.4%)	0 (0.0%)	6 (6.2%)	28 (12.8%)	8 (11.3%)	0.047	0.13
Intraretinal fluid, n (%)	2 (3.2%)	3 (3.8%)	4 (4.4%)	4 (5.4%)	0 (0.0%)	8 (8.4%)	42 (19.5%)	7 (9.9%)	0.93	0.007
Fibrosis, n (%)	3 (4.9%)	5 (6.4%)	10 (11.0%)	8 (10.8%)	1 (4.8%)	1 (1.0%)	24 (11.2%)	9 (12.7%)	0.45	0.01
Tubulations, n (%)	0 (0.0%)	11 (14.1%)	13 (14.3%)	10 (13.5%)	1 (4.8%)	2 (2.1%)	19 (8.8%)	3 (4.2%)	0.02	0.12

*BMI* body mass index, *SD* standard deviation, *B* Boston, *P* Portugal. P value is based on the ANOVA test for continuous variables and the chi-square test for categorical variables.

Significant values are in bold (P-value < 0.05).

Regarding retinal structure as assessed by OCT, the endotypes were significantly differed with respect to classic drusen (p = 3.29e–08), atrophy (p = 0.0009), EZ disruption (p = 3.65e–10), hyperreflective foci (p = 1.19e–07), subretinal fluid (p = 0.047), intraretinal fluid (p = 0.93), and tubulations (p = 0.02).

There was a significant difference in AMD stage across the metabo-endotypes (p = 2.95e–06), demonstrating a clear association between metabo-endotypes and disease stages. Endotype 1 predominantly consisted of Early AMD (22 cases, 66.7%), with Intermediate AMD (6 cases, 18.2%) and Late AMD (5 cases, 15.2%) also present. Endotype 2 was characterized by a higher proportion of Intermediate AMD (28 cases, 65.1%), but also included Early AMD (5 cases, 11.6%) and Late AMD (10 cases, 23.3%). Endotype 3 showed a predominance of Late AMD (21 cases, 44.7%), with Intermediate AMD (16 cases, 34.0%) and Early AMD (10 cases, 21.3%). Endotype 4, despite having the most severe dysfunction indicators based on RIT and AUDAC, had a notable percentage of Early AMD (18 cases, 45.0%), as well as Intermediate AMD (13 cases, 32.5%) and Late AMD (9 cases, 22.5%).

### Validation of metabo-endotypes in Coimbra, Portugal

Individuals within the same metabolomic-endotype across the two cohorts can be considered metabolomically equivalent, and therefore, we sought to determine if they also displayed similar phenotypic characteristics. The metabo-endotypes identified in the Boston cohort were recapitulated in the Portuguese study cohort, consisting of 11, 49, 116, and 38 cases for metabo-endotypes 1 to 4, respectively.

**Figure 2:**
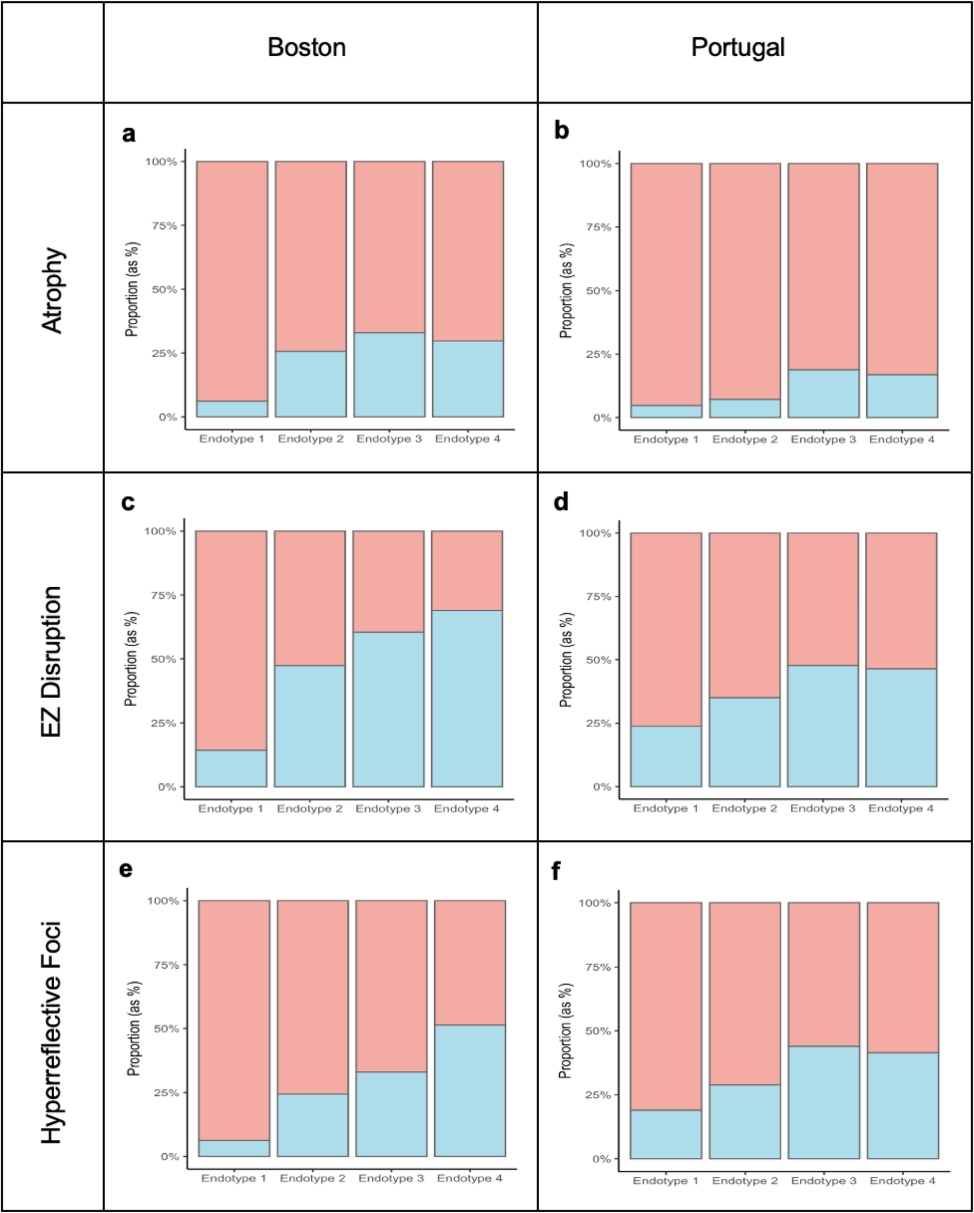
Stacked bar chart showing the proportion (as %) of atrophy, EZ disruption, and hyperreflective foci for boston (**a**,**c**,**e**) and Portugal (**b**,**d**,**f**) respectively. Red represents the proportion that doesn’t have the condition, while blue indicates the proportion that has it.

In the Portuguese validation cohort, significant associations were replicated for AMD stage (p = 0.02), atrophy (p = 0.03), EZ disruption (p = 0.045), hyperreflective foci (p = 0.02), intraretinal fluid (p = 0.007) and fibrosis (p = 0.01). A similar proportion of individuals with atrophy, EZ disruption, and hyperreflective foci for Boston, US and Coimbra, Portugal by metabolomic-endotype was observed, with Endotype 1 being showing the least prevalence of OCT findings and Endotypes 3 and 4 being the most (Fig. 2, Table 2). For Endotype 1, which showed the least prevalence of OCT findings, the proportions were as follows: In Boston, atrophy 6.2%, EZ disruption 14.3%, hyperreflective foci 6.2%; in Portugal, atrophy 4.8%, EZ disruption 23.8%, hyperreflective foci 19.0% (Fig. 2, Table 2). Conversely, endotypes 3 and 4 exhibited higher rates of these conditions. For Boston, the proportions were: atrophy 33.0% for Endotype 3 and 29.7% for Endotype 4, EZ disruption 60.4% for Endotype 3 and 68.9% for Endotype 4, hyperreflective foci 33.0% for Endotype 3 and 51.4% for Endotype 4 (Fig. 2, Table 2). Similar trends were observed in Portugal: atrophy 18.8% for Endotype 3 and 16.9% for Endotype 4, EZ disruption 47.8% for Endotype 3 and 46.5% for Endotype 4, hyperreflective foci 44.0% for Endotype 3 and 41.4% for Endotype 4 (Fig. 2, Table 2). These results show consistent associations between the identified metabo-endotypes and key clinical factors, in both the training and validation cohorts. As mentioned, DA testing was not available for this validation cohort, so these results were not possible to validate.

Significant differences in AMD stage were observed across metabo-endotypes in the Portuguese cohort (p = 0.02). For Endotype 1, the distribution was primarily Early AMD (2 cases, 18.2%), followed by Intermediate AMD (8 cases, 72.7%) and Late AMD (1 case, 9.1%). Endotype 2 displayed a predominance of Intermediate AMD (37 cases, 75.5%), with Early AMD (9 cases, 18.4%) and Late AMD (3 cases, 6.1%) also represented. In Endotypes 3 and 4, the distribution of AMD stages was similarly varied. Endotype 3 was characterized by a significant presence of Late AMD (38 cases, 32.8%), with a majority in Intermediate AMD (61 cases, 52.6%) and a smaller fraction in Early AMD (17 cases, 14.7%). Likewise, Endotype 4 also exhibited a diverse distribution across the stages: Late AMD (12 cases, 31.6%), the majority being Intermediate AMD (22 cases, 57.9%), and Early AMD (4 cases, 10.5%).

### Key metabolite metabolomic-endotype drivers

We then performed a meta-analysis of the metabolites identified for each endotype in the Boston, US and Coimbra, Portugal cohorts, and significant associations were identified between specific metabolites and each metabolomic-endotype. A total of 96, 173, 261, and 73 metabolites were significantly associated with metabo-endotypes 1, 2, 3, and 4, respectively based on ANOVA and post-hoc q-values < 0.05. These results are presented in Tables S1 to S4.

**Figure 3:**
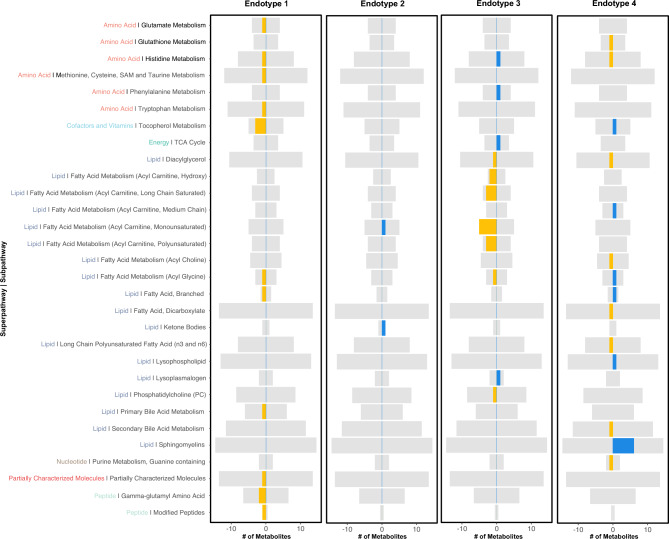
Distribution of metabolites across different endotypes by pathway: a visual representation of metabolites evaluated for their significance based on ANOVA q-values and post-hoc q-values. These metabolites are organized by super pathways and sub-pathways. Grey bars illustrate the potential total number of metabolites in the study. The yellow and blue indicators mark lower and higher mean values of metabolites for specific endotypes, respectively. The extent of these colored segments indicates the quantity of significant metabolites in each classification.

Endotype 1, characterized by a lower prevalence of OCT changes, was associated with low levels of amino acids in the glutamate, histidine, tryptophan and methionine, cysteine, SAM and taurine metabolism pathway (Fig. 3, Table S1). Endotype 2 was associated with increased levels of an acyl carnitine metabolite and a ketone body (acetoacetate) (Fig. 3, Table S2). Endotype 3, the second most associated with structural changes, was broadly associated with low levels of fatty acid metabolites (acyl carnitines) (Fig. 3, Table S3). Endotype 4, which was differed from the other metabo-endotypes with the highest number of phenotypic parameters was broadly associated with increased levels sphingomyelins and fatty acid metabolites (Fig. 3, Table S4). As shown, even though there was some crossover in the metabolites associated with each metabolomic-endotype, the direction of effect often differed. This is well exemplified by acyl carnitines that were broadly decreased in association with Endotype 2, while increased in association with Endotype 3.

## Discussion

Traditional classifications of AMD have historically depended on color photography to assess disease progression by measuring the size and number of drusen, implying a linear progression from early to intermediate, and ultimately to late stages, without distinguishing any specific subtypes^26^. This study introduces a nuanced approach, establishing a pathophysiological basis for categorizing AMD into four putative metabo-endotypes. This categorization leverages assessments of both retinal function (RIT and AUDAC) and structure (atrophy, EZ disruption, and hyperreflective foci), with Endotype 1 encompassing primarily early-stage patients, Endotype 2 those in the intermediate stage, and Endotypes 3 and 4 representing more advanced disease states. Interestingly, within the Boston cohort, Endotype 4 consists of a significant fraction of Early AMD patients (45.0%), challenging the straightforward correlation between endotypes and conventional disease stages. This suggests that metabolomics offers a more precise pathophysiological differentiation, advancing beyond traditional evaluation methods by illustrating a dynamic disease progression across the metabo-endotypes.

Despite similar retinal impairments, Endotypes 3 and 4 reveal distinct pathological pathways within the same advanced stage of AMD, shedding light on the disease's multifaceted nature. This distinction suggests the development of two unique subtypes in the later disease phases, defined by specific pathophysiological traits, underscoring metabolomics' role in enhancing our comprehension of AMD beyond traditional models. This refined classification not only challenges but also enriches existing paradigms by suggesting that AMD's linear progression model can coexist with a sophisticated metabo-endotype framework, offering a comprehensive understanding of the disease.

The major metabolomic drivers of Endotype 3 were fatty acid metabolites, specifically acyl carnitines. Acyl carnitines play a crucial role in mitochondrial fatty acid metabolism and energy production^27^. In particular, they transport acyl groups from fatty acids and branched-chain amino acids into mitochondria to generate cellular energy. Interestingly, dysregulations in the levels of these metabolites have been reported in Alzheimer’s disease^28^, which shares known common pathways with AMD, and more recently in the vitreous of patients with intermediate AMD^29^. And also the plasma of patients with neovascular AMD compared to controls^30^. These data support an important role for these metabolites in the pathophysiology of more advanced subtypes of AMD, likely related to their associations with oxidative stress. Of note, Endotype 3 also encompassed changes in a small number of amino acids, one of them N-acetylphenylalanine. This is particularly interesting as dysregulations of this metabolite have been consistently reported in AMD both by our group and others^10^.

Even though Endotype 4 also showed some associations with fatty acid metabolites (acyl carnitines), there was also a considerable number of associations seen with sphingomyelins. Sphingomyelins are sphingolipids that serve as key mediators of inflammation and cell death, participating in signaling pathways involved in apoptosis, autophagy and stress responses^31^. In the retina, elevated levels of sphingomyelins and other sphingolipids have been linked to degeneration^32^ and advanced AMD^33^, and has led to apoptosis of the RPE and photoreceptors via oxidative stress^34^. Interestingly, the common genetic variant rs1061170 in *CFH*, which is a strong risk factor for AMD, seems to influence the levels of some sphingomyelins in the serum^33^.

Endotype 1 was associated with low levels of amino acids in multiple pathways. Among them, beta-citrylglutamate, an amino acid that participates in the glutamate metabolism pathway. Dysregulations in the glutamate pathway, a neurotransmitter^40^, that participates in mitochondrial energy metabolism, have been consistently reported in AMD both by our group^9,14^, and a recent study by the Eye-Risk consortium that showed that glutamine and glutaminolysis were among the most predictive features to discriminate between patients with non-advanced AMD and controls^36^. Endotype 1 also included dysregulations of kynurenate, a metabolite of the tryptophan metabolism pathway, that is known to influence the activity of glutamate both via direct and indirect effects^35^. Interestingly, kynurenate also participates in the synthesis of NAD + and has anti-inflammatory roles^36^. Inflammation plays an important role in AMD pathogenesis^37^. An amino acid part of the methionine, cysteine, SAM and taurine metabolism was also dysregulated in Endotype 1. The taurine metabolism pathway has also been previously associated with retinal health, as taurine is a critical amino acid for photoreceptor function and retinal development^38^.

This study acknowledges several limitations that warrant consideration. Firstly, the sample size is relatively small, which may limit the generalizability of the findings. Additionally, the absence of dark adaptation testing in our Portuguese cohort presents a methodological gap. The identification of four metabo-endotypes introduces a novel challenge to the conventional staging of AMD (Early, Intermediate, and Late), suggesting the traditional classification may underestimate the disease's complexity. The possibility that a larger sample could reveal five or more distinct metabo-endotypes underscores the need for a more nuanced understanding of AMD's pathophysiology. The ideal scenario would involve enrolling patients at the same disease stage, ideally early in the diagnosis, to more accurately track disease progression. However, the constraints of our sample size made this approach unfeasible. Moreover, this cross-sectional study design leaves room for longitudinal studies to explore how metabo-endotypes correlate with AMD's progression over time. While we assessed structural parameters in a binary manner, future studies could benefit from more detailed quantification.

Despite these limitations, our study stands out for its innovative approach, employing a bottom-up methodology that correlates molecular signatures with clinical endotypes, supported by the use of four metabolomic profiling platforms. These platforms offer an extensive overview of the metabolome, although they do not capture its entirety. Future research could expand this by incorporating additional methods like gas chromatography-mass spectrometry (GC–MS) or nuclear magnetic resonance (NMR) to encompass a wider range of metabolites. Integrating other 'omics' disciplines may also enhance our understanding of AMD's pathophysiology. While utilizing plasma metabolome data provides insights into systemic levels, it's important to acknowledge that AMD primarily affects the eyes, suggesting that local ocular changes might not be fully captured by this method. Direct metabolomic analysis of ocular tissues could offer more precise insights into the local pathophysiological changes. Additionally, we used a wide refractive error exclusion criteria of + 6D/–6D, aimed at excluding individuals with severe myopia or hyperopia. This criterion was established to focus our research scope, despite the known variations in metabolomic profiles of aqueous/vitreous humor between myopia and emmetropia^39,40^, suggesting an area for further investigation.

To our knowledge, this study is the first to apply metabolomics to AMD endotyping and use an unsupervised metabolome-wide clustering approach. We identified and validated in an independent cohort four putative metabo-driven AMD endotypes. Characterized by unique metabolite profiles, these endotypes suggest divergent pathophysiological pathways across different stages of the disease. Notably, our findings reveal potential subtypes within the more severe forms of AMD, highlighting the complexity and variability in disease progression. Further research is needed to elucidate the precise role of these metabolites in AMD and their potential as biomarkers or treatment targets. We believe that these findings offer insight into the underlying mechanisms of AMD and open the door for personalized approaches to managing this blinding disease.

### Supplementary Information

Supplementary Tables.

## Data Availability

The data that support the findings of this study are available from the corresponding author upon reasonable request.

## References

[1] Wong WL (2014). Global prevalence of age-related macular degeneration and disease burden projection for 2020 and 2040: A systematic review and meta-analysis. Lancet Glob. Health.

[2] Yonekawa Y, Miller JW, Kim IK (2015). Age-related macular degeneration: Advances in management and diagnosis. J. Clin. Med..

[3] Sobrin L, Seddon JM (2014). Nature and nurture- genes and environment- predict onset and progression of macular degeneration. Prog. Retin Eye Res..

[4] Spaide RF (2018). Improving the age-related macular degeneration construct: A new classification system. Retina.

[5] Chen Y, Bedell M, Zhang K (2010). Age-related macular degeneration: Genetic and environmental factors of disease. Mol. Interv..

[6] Nicholson JK, Lindon JC, Holmes E (1999). 'Metabonomics': Understanding the metabolic responses of living systems to pathophysiological stimuli via multivariate statistical analysis of biological NMR spectroscopic data. Xenobiotica.

[7] Kelly RS (2022). Metabo-endotypes of asthma reveal differences in lung function: Discovery and validation in two TOPMed cohorts. Am. J. Respir. Crit. Care Med..

[8] Tyler SR, Bunyavanich S (2019). Leveraging -omics for asthma endotyping. J. Allergy Clin. Immunol..

[9] Brown CN (2018). Metabolomics and age-related macular degeneration. Metabolites.

[10] Hou X-W, Wang Y, Pan C-W (2020). Metabolomics in age-related macular degeneration: A systematic review. Investig. Ophthalmol. Vis. Sci..

[11] Laíns I (2019). Human plasma metabolomics in age-related macular degeneration: Meta-analysis of two cohorts. Metabolites.

[12] Laíns I (2017). Human plasma metabolomics in age-related macular degeneration (AMD) using nuclear magnetic resonance spectroscopy. PLoS One.

[13] Laíns I (2018). Human plasma metabolomics study across all stages of age-related macular degeneration identifies potential lipid biomarkers. Ophthalmology.

[14] Mendez KM (2021). Association of human plasma metabolomics with delayed dark adaptation in age-related macular degeneration. Metabolites.

[15] Tsikata E (2017). Automated brightness and contrast adjustment of color fundus photographs for the grading of age-related macular degeneration. Transl. Vis. Sci. Technol..

[16] Age-Related Eye Disease Study Research Group (2001). The Age-Related Eye Disease Study system for classifying age-related macular degeneration from stereoscopic color fundus photographs: The Age-Related Eye Disease Study Report Number 6. Am. J. Ophthalmol..

[17] Danis RP (2013). Methods and reproducibility of grading optimized digital color fundus photographs in the age-related eye disease Study 2 (AREDS2 Report Number 2). Investig. Ophthalmol. Vis. Sci..

[18] Laíns I (2019). Urine nuclear magnetic resonance (NMR) metabolomics in age-related macular degeneration. J. Proteome Res..

[19] Ford L (2020). Precision of a clinical metabolomics profiling platform for use in the identification of inborn errors of metabolism. J. Appl. Lab. Med..

[20] Wang B (2014). Similarity network fusion for aggregating data types on a genomic scale. Nat. Methods.

[21] Li CX, Wheelock CE, Sköld CM, Wheelock Å, M.  (2018). Integration of multi-omics datasets enables molecular classification of COPD. Eur. Respir. J..

[22] Pearl J (1988). Probabilistic Reasoning in Intelligent Systems: Networks of Plausible Inference.

[23] Ng A, Jordan M, Weiss Y (2001). On spectral clustering: Analysis and an algorithm. Adv. Neural Inf. Process. Syst..

[24] Zelnik-Manor L, Perona P (2004). Self-tuning spectral clustering. Adv. Neural Inf. Process. Syst..

[25] Zhu, X. & Ghahramani, Z. Learning from labeled and unlabeled data with label propagation (2002).

[26] Lim LS, Mitchell P, Seddon JM, Holz FG, Wong TY (2012). Age-related macular degeneration. Lancet.

[27] Fliesler SJ, Bretillon L (2010). The ins and outs of cholesterol in the vertebrate retina. J. Lipid Res..

[28] Cristofano A (2016). Serum levels of acyl-carnitines along the continuum from normal to Alzheimer’s dementia. PLoS One.

[29] Yoon CK (2023). Vitreous fatty amides and acyl carnitines are altered in intermediate age-related macular degeneration. Investig. Ophthalmol. Vis. Sci..

[30] Liew G (2020). Acylcarnitine abnormalities implicate mitochondrial dysfunction in patients with neovascular age-related macular degeneration. Investig. Ophthalmol. Vis. Sci..

[31] Shiwani HA (2021). Updates on sphingolipids: Spotlight on retinopathy. Biomed. Pharmacother..

[32] van Leeuwen EM (2018). A new perspective on lipid research in age-related macular degeneration. Prog. Retinal Eye Res..

[33] Pujol-Lereis LM (2018). Evaluation of serum sphingolipids and the influence of genetic risk factors in age-related macular degeneration. PLoS One.

[34] Mondal K, Mandal N (2019). Role of bioactive sphingolipids in inflammation and eye diseases. Adv. Exp. Med. Biol..

[35] Schwarcz R (2016). Kynurenines and glutamate: Multiple links and therapeutic implications. Adv. Pharmacol..

[36] Stone TW (2022). An integrated cytokine and kynurenine network as the basis of neuroimmune communication. Front. Neurosci..

[37] Wang Y, Wang VM, Chan CC (2011). The role of anti-inflammatory agents in age-related macular degeneration (AMD) treatment. Eye.

[38] Heller-Stilb B (2002). Disruption of the taurine transporter gene (taut) leads to retinal degeneration in mice. Faseb J..

[39] Grochowski ET (2020). Omics in myopia. J. Clin. Med..

[40] Hou X-W, Wang Y, Ke C, Pan C-W (2023). Metabolomics facilitates the discovery of metabolic profiles and pathways for myopia: A systematic review. Eye.

