# Supplementary material for: Metabolomic-derived endotypes of age-related macular degeneration (AMD): a step towards identification of disease subgroups
Source: Sci Rep. 2024 May 27;14:12145. doi: 10.1038/s41598-024-59045-z (PMC11130126; doi:10.1038/s41598-024-59045-z)
Supplement: Supplementary file 1 — Supplementary Tables. [file 41598_2024_59045_MOESM1_ESM.docx]

**Supplementary Material**

**Metabolomic Endotypes of Age-related Macular Degeneration (AMD) using Machine Learning: A Step Towards Precision Medicine**

Kevin Mendez MS, Ines Lains MD PhD, Rachel S. Kelly PhD , João Gil MD, Rufino Silva MD PhD, John Miller MD, Demetrios G. Vavvas MD PhD, Ivana Kim MD, Joan Miller MD , Liming Liang PhD, Jessica A. Lasky-Su ScD, and Deeba Husain MD

**Table of contents of PDF file**

Table S1: Metabolites significantly associated with Endotype 1 based on ANOVA q-value < 0.05 and post-hoc q-values < 0.05.

Table S2. Metabolites significantly associated with Endotype 2 based on ANOVA q-value < 0.05 and post-hoc q-values < 0.05.

Table S3: Metabolites significantly associated with Endotype 3 based on ANOVA q-value < 0.05 and post-hoc q-values < 0.05.

Table S4: Metabolites significantly associated with Endotype 4 based on ANOVA q-value < 0.05 and post-hoc q-values < 0.05.

**Table S1. Metabolites significantly associated with Endotype 1 based on ANOVA q-value < 0.05 and post-hoc q-values < 0.05.**

| **Name** | **Super-Pathway** | **Sub-Pathway** | **Endotype 1 Mean (SD)**  **[B** \| **P]** | **Endotype 2 Mean (SD)**  **[B** \| **P]** | **Endotype 3 Mean (SD)**  **[B** \| **P]** | **Endotype 4 Mean (SD)**  **[B** \| **P]** | **ANOVA P-Value** | **ANOVA Q-Value** |
| --- | --- | --- | --- | --- | --- | --- | --- | --- |
| beta-citrylglutamate | Amino Acid | Glutamate Metabolism | -0.11(0.16) \| -0.08(0.15) | 0.00(0.13) \| 0.06(0.21) | 0.01(0.18) \| 0.00(0.24) | 0.07(0.17) \| 0.00(0.21) | 0.0004 | 0.001 |
| N-acetyl-3-methylhistidine* | Amino Acid | Histidine Metabolism | -0.45(0.47) \| -0.13(0.53) | 0.05(0.38) \| 0.01(0.56) | 0.19(0.51) \| 0.07(0.55) | 0.09(0.54) \| 0.03(0.53) | 1.34E-06 | 1.01E-05 |
| S-adenosylhomocysteine (SAH) | Amino Acid | Methionine, Cysteine, SAM and Taurine Metabolism | -0.12(0.21) \| -0.12(0.23) | 0.02(0.10) \| 0.07(0.21) | 0.08(0.16) \| 0.01(0.25) | -0.02(0.20) \| 0.02(0.21) | 3.36E-05 | 0.0002 |
| kynurenate | Amino Acid | Tryptophan Metabolism | -0.14(0.15) \| 0.00(0.13) | 0.01(0.14) \| 0.01(0.15) | 0.05(0.16) \| 0.00(0.16) | 0.05(0.19) \| 0.00(0.15) | 1.10E-05 | 5.86E-05 |
| gamma-CEHC glucuronide* | Cofactors and Vitamins | Tocopherol Metabolism | -0.44(0.40) \| -0.01(0.48) | -0.02(0.38) \| -0.07(0.46) | 0.16(0.44) \| -0.02(0.37) | 0.20(0.44) \| 0.08(0.44) | 5.43E-09 | 8.81E-08 |
| delta-CEHC | Cofactors and Vitamins | Tocopherol Metabolism | -0.45(0.33) \| 0.04(0.48) | -0.02(0.42) \| 0.00(0.45) | 0.09(0.42) \| -0.05(0.39) | 0.28(0.40) \| 0.02(0.42) | 1.52E-10 | 4.09E-09 |
| delta-CEHC glucuronide | Cofactors and Vitamins | Tocopherol Metabolism | -0.35(0.14) \| 0.04(0.26) | 0.04(0.39) \| 0.00(0.22) | 0.16(0.43) \| -0.03(0.16) | 0.06(0.40) \| 0.00(0.23) | 6.43E-07 | 5.22E-06 |
| cis-3,4-methyleneheptanoylglycine | Lipid | Fatty Acid Metabolism (Acyl Glycine) | -0.43(0.49) \| 0.03(0.48) | -0.02(0.26) \| -0.02(0.46) | -0.02(0.38) \| -0.01(0.42) | 0.39(0.28) \| 0.01(0.48) | 4.61E-15 | 4.49E-13 |
| cis-3,4-methyleneheptanoate | Lipid | Fatty Acid, Branched | -0.44(0.44) \| 0.06(0.39) | -0.10(0.31) \| -0.04(0.47) | -0.02(0.41) \| -0.02(0.46) | 0.49(0.25) \| 0.01(0.48) | 3.76E-19 | 1.46E-16 |
| taurocholate | Lipid | Primary Bile Acid Metabolism | -0.62(0.38) \| 0.07(0.67) | 0.08(0.61) \| 0.07(0.69) | 0.23(0.78) \| -0.06(0.67) | 0.16(0.82) \| -0.06(0.60) | 4.52E-06 | 2.81E-05 |
| glycine conjugate of C10H14O2 (1)* | Partially Characterized Molecules | Partially Characterized Molecules | -0.50(0.50) \| 0.06(0.52) | 0.02(0.25) \| 0.00(0.44) | 0.04(0.4) \| -0.04(0.38) | 0.34(0.43) \| 0.00(0.53) | 4.20E-13 | 2.34E-11 |
| gamma-glutamylleucine | Peptide | Gamma-glutamyl Amino Acid | -0.07(0.08) \| -0.02(0.09) | 0.01(0.07) \| 0.01(0.09) | 0.02(0.09) \| 0.00(0.09) | 0.02(0.10) \| 0.01(0.10) | 0.0001 | 0.0005 |
| gamma-glutamylisoleucine* | Peptide | Gamma-glutamyl Amino Acid | -0.12(0.13) \| -0.03(0.13) | 0.00(0.10) \| 0.02(0.13) | 0.06(0.13) \| 0.00(0.12) | 0.03(0.13) \| 0.01(0.14) | 4.42E-08 | 5.55E-07 |

Definition of abbreviations: SD = Standard Deviation; B = Boston; P = Portugal.

**Table S2. Metabolites significantly associated with Endotype 2 based on ANOVA q-value < 0.05 and post-hoc q-values < 0.05.**

| **Name** | **Super-Pathway** | **Sub-Pathway** | **Endotype 1 Mean (SD)**  **[B** \| **P]** | **Endotype 2 Mean (SD)**  **[B** \| **P]** | **Endotype 3 Mean (SD)**  **[B** \| **P]** | **Endotype 4 Mean (SD)**  **[B** \| **P]** | **ANOVA P-Value** | **ANOVA Q-Value** |
| --- | --- | --- | --- | --- | --- | --- | --- | --- |
| 3-decenoylcarnitine | Lipid | Fatty Acid Metabolism (Acyl Carnitine, Monounsaturated) | -0.22(0.38) \| 0.01(0.44) | 0.39(0.37) \| 0.03(0.48) | 0.06(0.38) \| -0.04(0.43) | -0.30(0.43) \| -0.01(0.46) | 1.63E-12 | 6.69E-11 |
| acetoacetate | Lipid | Ketone Bodies | -0.21(0.44) \| -0.03(0.35) | 0.44(0.43) \| 0.02(0.43) | -0.04(0.39) \| -0.06(0.35) | -0.26(0.39) \| 0.06(0.41) | 9.28E-12 | 3.01E-10 |

Definition of abbreviations: SD = Standard Deviation; B = Boston; P = Portugal.

**Table S3. Metabolites significantly associated with Endotype 3 based on ANOVA q-value < 0.05 and post-hoc q-values < 0.05.**

| **Name** | **Super-Pathway** | **Sub-Pathway** | **Endotype 1 Mean (SD)**  **[B** \| **P]** | **Endotype 2 Mean (SD)**  **[B** \| **P]** | **Endotype 3 Mean (SD)**  **[B** \| **P]** | **Endotype 4 Mean (SD)**  **[B** \| **P]** | **ANOVA P-Value** | **ANOVA Q-Value** |
| --- | --- | --- | --- | --- | --- | --- | --- | --- |
| 1-ribosyl-imidazoleacetate* | Amino Acid | Histidine Metabolism | -0.08(0.13) \| 0.01(0.17) | -0.04(0.15) \| -0.01(0.21) | 0.13(0.18) \| -0.02(0.15) | -0.05(0.16) \| 0.02(0.17) | 4.59E-08 | 5.68E-07 |
| N-acetylphenylalanine | Amino Acid | Phenylalanine Metabolism | -0.07(0.12) \| -0.02(0.14) | -0.04(0.12) \| 0.01(0.20) | 0.10(0.14) \| 0.01(0.17) | -0.02(0.12) \| 0.00(0.16) | 6.37E-08 | 7.52E-07 |
| succinylcarnitine (C4-DC) | Energy | TCA Cycle | -0.08(0.16) \| 0.01(0.21) | -0.01(0.09) \| -0.05(0.21) | 0.09(0.12) \| 0.04(0.15) | -0.03(0.14) \| 0.00(0.16) | 1.86E-06 | 1.31E-05 |
| palmitoyl-oleoyl-glycerol (16:0/18:1) [1]* | Lipid | Diacylglycerol | 0.06(0.46) \| 0.01(0.42) | 0.13(0.46) \| 0.00(0.44) | -0.33(0.44) \| 0.03(0.42) | 0.19(0.39) \| -0.03(0.46) | 1.25E-06 | 9.47E-06 |
| 3-hydroxypalmitoylcarnitine | Lipid | Fatty Acid Metabolism (Acyl Carnitine, Hydroxy) | -0.03(0.22) \| 0.05(0.13) | 0.13(0.25) \| 0.00(0.22) | -0.26(0.32) \| -0.02(0.16) | 0.18(0.14) \| -0.01(0.17) | 9.48E-14 | 8.21E-12 |
| 3-hydroxyoleoylcarnitine | Lipid | Fatty Acid Metabolism (Acyl Carnitine, Hydroxy) | 0.02(0.25) \| 0.02(0.21) | 0.24(0.22) \| 0.03(0.26) | -0.33(0.34) \| -0.08(0.26) | 0.12(0.21) \| 0.02(0.19) | 2.06E-17 | 4.58E-15 |
| palmitoylcarnitine (C16) | Lipid | Fatty Acid Metabolism (Acyl Carnitine, Long Chain Saturated) | 0.01(0.11) \| 0.01(0.11) | 0.12(0.13) \| 0.03(0.12) | -0.17(0.24) \| -0.01(0.11) | 0.06(0.11) \| -0.02(0.12) | 5.94E-13 | 2.72E-11 |
| myristoylcarnitine (C14) | Lipid | Fatty Acid Metabolism (Acyl Carnitine, Long Chain Saturated) | -0.01(0.17) \| 0.02(0.14) | 0.13(0.18) \| 0.03(0.22) | -0.22(0.28) \| -0.01(0.16) | 0.13(0.12) \| -0.03(0.17) | 1.76E-13 | 1.14E-11 |
| margaroylcarnitine (C17)* | Lipid | Fatty Acid Metabolism (Acyl Carnitine, Long Chain Saturated) | 0.00(0.13) \| 0.02(0.13) | 0.07(0.17) \| 0.02(0.13) | -0.17(0.25) \| 0.00(0.12) | 0.13(0.13) \| -0.03(0.13) | 4.14E-11 | 1.24E-09 |
| oleoylcarnitine (C18:1) | Lipid | Fatty Acid Metabolism (Acyl Carnitine, Monounsaturated) | 0.03(0.11) \| 0.00(0.13) | 0.15(0.15) \| 0.04(0.15) | -0.20(0.26) \| -0.04(0.14) | 0.04(0.15) \| -0.01(0.15) | 1.53E-13 | 1.11E-11 |
| myristoleoylcarnitine (C14:1)* | Lipid | Fatty Acid Metabolism (Acyl Carnitine, Monounsaturated) | 0.00(0.19) \| -0.01(0.22) | 0.22(0.24) \| 0.05(0.29) | -0.23(0.30) \| -0.04(0.23) | 0.03(0.19) \| 0.00(0.24) | 2.57E-13 | 1.54E-11 |
| palmitoleoylcarnitine (C16:1)* | Lipid | Fatty Acid Metabolism (Acyl Carnitine, Monounsaturated) | 0.01(0.14) \| 0.01(0.15) | 0.18(0.19) \| 0.05(0.20) | -0.21(0.24) \| -0.04(0.16) | 0.05(0.14) \| -0.01(0.18) | 5.51E-16 | 7.16E-14 |
| ximenoylcarnitine (C26:1)* | Lipid | Fatty Acid Metabolism (Acyl Carnitine, Monounsaturated) | 0.01(0.12) \| 0.02(0.15) | 0.11(0.14) \| 0.02(0.13) | -0.15(0.25) \| -0.03(0.13) | 0.04(0.13) \| -0.01(0.16) | 2.60E-09 | 4.70E-08 |
| eicosenoylcarnitine (C20:1)* | Lipid | Fatty Acid Metabolism (Acyl Carnitine, Monounsaturated) | 0.02(0.16) \| 0.01(0.14) | 0.12(0.16) \| 0.04(0.15) | -0.18(0.31) \| -0.04(0.16) | 0.07(0.16) \| 0.00(0.17) | 7.94E-09 | 1.22E-07 |
| arachidonoylcarnitine (C20:4) | Lipid | Fatty Acid Metabolism (Acyl Carnitine, Polyunsaturated) | 0.01(0.14) \| 0.01(0.14) | 0.13(0.20) \| 0.04(0.18) | -0.19(0.32) \| -0.02(0.16) | 0.07(0.19) \| -0.03(0.17) | 1.98E-08 | 2.80E-07 |
| dihomo-linoleoylcarnitine (C20:2)* | Lipid | Fatty Acid Metabolism (Acyl Carnitine, Polyunsaturated) | 0.03(0.18) \| 0.01(0.17) | 0.16(0.16) \| 0.04(0.15) | -0.20(0.31) \| -0.03(0.15) | 0.03(0.19) \| -0.02(0.19) | 6.38E-10 | 1.38E-08 |
| dihomo-linolenoylcarnitine (C20:3n3 or 6)* | Lipid | Fatty Acid Metabolism (Acyl Carnitine, Polyunsaturated) | 0.03(0.16) \| 0.01(0.14) | 0.11(0.17) \| 0.05(0.18) | -0.18(0.30) \| -0.02(0.14) | 0.07(0.16) \| -0.03(0.18) | 8.74E-09 | 1.31E-07 |
| hexanoylglycine | Lipid | Fatty Acid Metabolism (Acyl Glycine) | 0.11(0.32) \| 0.04(0.33) | 0.17(0.38) \| -0.01(0.37) | -0.27(0.38) \| -0.04(0.40) | 0.05(0.37) \| 0.01(0.36) | 1.39E-06 | 1.03E-05 |
| 1-(1-enyl-palmitoyl)-GPE (P-16:0)* | Lipid | Lysoplasmalogen | -0.03(0.16) \| -0.06(0.14) | -0.05(0.21) \| 0.02(0.16) | 0.12(0.29) \| 0.02(0.18) | -0.06(0.10) \| 0.01(0.17) | 0.0003 | 0.0008 |
| 1-stearoyl-2-arachidonoyl-GPC (18:0/20:4) | Lipid | Phosphatidylcholine (PC) | -0.01(0.08) \| 0.02(0.08) | 0.04(0.07) \| -0.02(0.08) | -0.10(0.16) \| 0.00(0.08) | 0.07(0.07) \| 0.01(0.07) | 1.91E-10 | 4.96E-09 |

Definition of abbreviations: SD = Standard Deviation; B = Boston; P = Portugal.

**Table S4. Metabolites significantly associated with Endotype 4 based on ANOVA q-value < 0.05 and post-hoc q-values < 0.05.**

| **Name** | **Super-Pathway** | **Sub-Pathway** | **Endotype 1 Mean (SD)**  **[B** \| **P]** | **Endotype 2 Mean (SD)**  **[B** \| **P]** | **Endotype 3 Mean (SD)**  **[B** \| **P]** | **Endotype 4 Mean (SD)**  **[B** \| **P]** | **ANOVA P-Value** | **ANOVA Q-Value** |
| --- | --- | --- | --- | --- | --- | --- | --- | --- |
| cysteine-glutathione disulfide | Amino Acid | Glutathione Metabolism | 0.12(0.21) \| -0.08(0.35) | 0.03(0.29) \| 0.00(0.36) | 0.12(0.19) \| 0.02(0.31) | -0.27(0.37) \| 0.04(0.32) | 2.46E-09 | 4.68E-08 |
| 4-imidazoleacetate | Amino Acid | Histidine Metabolism | 0.07(0.41) \| 0.01(0.52) | 0.11(0.23) \| -0.07(0.55) | 0.16(0.33) \| 0.07(0.48) | -0.37(0.53) \| -0.01(0.55) | 2.31E-08 | 3.15E-07 |
| gamma-CEHC | Cofactors and Vitamins | Tocopherol Metabolism | -0.25(0.32) \| 0.02(0.29) | -0.12(0.35) \| -0.04(0.33) | 0.04(0.34) \| -0.05(0.25) | 0.29(0.28) \| 0.05(0.31) | 1.58E-09 | 3.07E-08 |
| linoleoyl-docosahexaenoyl-glycerol (18:2/22:6) [2]* | Lipid | Diacylglycerol | 0.06(0.44) \| 0.05(0.46) | 0.23(0.36) \| 0.05(0.50) | 0.03(0.43) \| -0.07(0.54) | -0.33(0.40) \| -0.02(0.45) | 5.20E-07 | 4.35E-06 |
| cis-3,4-methyleneheptanoylcarnitine | Lipid | Fatty Acid Metabolism (Acyl Carnitine, Medium Chain) | -0.35(0.30) \| 0.03(0.28) | -0.05(0.28) \| 0.02(0.33) | -0.12(0.49) \| 0.00(0.27) | 0.48(0.27) \| -0.03(0.33) | 3.43E-17 | 5.35E-15 |
| docosahexaenoylcholine | Lipid | Fatty Acid Metabolism (Acyl Choline) | 0.04(0.25) \| -0.02(0.18) | 0.11(0.20) \| 0.06(0.19) | 0.04(0.22) \| -0.04(0.18) | -0.20(0.27) \| 0.00(0.18) | 5.46E-07 | 4.53E-06 |
| cis-3,4-methyleneheptanoylglycine | Lipid | Fatty Acid Metabolism (Acyl Glycine) | -0.43(0.49) \| 0.03(0.48) | -0.02(0.26) \| -0.02(0.46) | -0.02(0.38) \| -0.01(0.42) | 0.39(0.28) \| 0.01(0.48) | 4.61E-15 | 4.49E-13 |
| cis-3,4-methyleneheptanoate | Lipid | Fatty Acid, Branched | -0.44(0.44) \| 0.06(0.39) | -0.10(0.31) \| -0.04(0.47) | -0.02(0.41) \| -0.02(0.46) | 0.49(0.25) \| 0.01(0.48) | 3.76E-19 | 1.46E-16 |
| azelate (C9-DC) | Lipid | Fatty Acid, Dicarboxylate | 0.18(0.16) \| -0.07(0.15) | 0.22(0.25) \| 0.04(0.16) | 0.09(0.28) \| -0.01(0.17) | -0.49(0.40) \| 0.02(0.14) | 3.51E-22 | 2.73E-19 |
| docosahexaenoate (DHA; 22:6n3) | Lipid | Long Chain Polyunsaturated Fatty Acid (n3 and n6) | 0.05(0.17) \| -0.02(0.20) | 0.10(0.15) \| 0.06(0.21) | 0.04(0.21) \| -0.01(0.18) | -0.20(0.27) \| -0.03(0.2) | 9.71E-09 | 1.43E-07 |
| 1-linoleoyl-GPA (18:2)* | Lipid | Lysophospholipid | -0.13(0.26) \| -0.05(0.30) | -0.07(0.28) \| -0.04(0.28) | -0.11(0.33) \| 0.06(0.32) | 0.31(0.29) \| 0.02(0.30) | 4.04E-10 | 9.54E-09 |
| deoxycholic acid glucuronide | Lipid | Secondary Bile Acid Metabolism | 0.22(0.48) \| -0.02(0.50) | 0.12(0.45) \| 0.01(0.40) | 0.04(0.48) \| 0.01(0.41) | -0.35(0.50) \| 0.00(0.53) | 1.32E-05 | 6.93E-05 |
| sphingomyelin (d18:1/14:0, d16:1/16:0)* | Lipid | Sphingomyelins | -0.05(0.09) \| 0.00(0.11) | -0.02(0.10) \| 0.00(0.11) | -0.04(0.12) \| 0.00(0.11) | 0.11(0.09) \| -0.01(0.11) | 7.01E-10 | 1.48E-08 |
| sphingomyelin (d18:1/20:0, d16:1/22:0)* | Lipid | Sphingomyelins | -0.05(0.08) \| 0.00(0.09) | -0.02(0.07) \| -0.01(0.10) | -0.02(0.08) \| 0.02(0.08) | 0.09(0.08) \| -0.01(0.09) | 2.44E-12 | 9.06E-11 |
| sphingomyelin (d17:1/16:0, d18:1/15:0, d16:1/17:0)* | Lipid | Sphingomyelins | -0.04(0.11) \| 0.00(0.12) | -0.01(0.12) \| -0.01(0.12) | -0.05(0.12) \| 0.01(0.11) | 0.10(0.10) \| 0.00(0.11) | 4.85E-08 | 5.90E-07 |
| sphingomyelin (d18:1/21:0, d17:1/22:0, d16:1/23:0)* | Lipid | Sphingomyelins | -0.08(0.15) \| 0.00(0.15) | 0.00(0.13) \| -0.01(0.14) | -0.08(0.17) \| 0.01(0.13) | 0.15(0.13) \| 0.00(0.14) | 5.55E-11 | 1.54E-09 |
| sphingomyelin (d18:1/19:0, d19:1/18:0)* | Lipid | Sphingomyelins | -0.04(0.14) \| 0.02(0.14) | -0.01(0.14) \| -0.02(0.16) | -0.08(0.15) \| 0.02(0.15) | 0.12(0.13) \| -0.01(0.15) | 5.04E-08 | 6.04E-07 |
| sphingomyelin (d18:2/18:1)* | Lipid | Sphingomyelins | -0.02(0.09) \| 0.01(0.14) | -0.03(0.13) \| -0.02(0.11) | -0.05(0.12) \| 0.00(0.13) | 0.10(0.13) \| 0.01(0.13) | 4.10E-07 | 3.59E-06 |
| guanine | Nucleotide | Purine Metabolism, Guanine containing | 0.10(0.34) \| -0.15(0.40) | 0.05(0.23) \| 0.13(0.46) | 0.08(0.28) \| 0.02(0.47) | -0.23(0.35) \| -0.02(0.51) | 9.07E-06 | 4.98E-05 |

Definition of abbreviations: SD = Standard Deviation; B = Boston; P = Portugal.
